# Role of pharmacists in providing information on cancer genome medicine and patient decision making: Open-label randomized controlled study

**DOI:** 10.1016/j.rcsop.2026.100814

**Published:** 2026-06-18

**Authors:** Orie Saigo, Shuko Nojiri, Kota Asakura, Shoji Koshiba, Uki Saito, Satoshi Iwakawa, Toshimi Kimura, Shunsuke Kato

**Affiliations:** a Department of Clinical Oncology, Juntendo University Graduate School of Medicine, Tokyo, Japan; b Department of Pharmacy, Juntendo University Hospital, Tokyo, Japan; c Medical Technology Innovation Center, Juntendo University, Tokyo, Japan; d Department of Pharmacy, Juntendo University Urayasu Hospital, Chiba, Japan

**Keywords:** Cancer genome medicine, Precision medicine, Comprehensive genome profiling, Pharmacist, Decision making, Information provision

## Abstract

Cancer genome medicine grounded in genetic alterations is advancing worldwide, with comprehensive genome profiling (CGP) now routinely implemented in Japan. Although the role of pharmacists in cancer genome medicine remains unclear, they may serve as trusted sources of CGP-related information, potentially enhancing patient health literacy and supporting informed decision making. This study evaluated whether pharmacists providing CGP information earlier than the completion of standard therapy affects patient decision making. Using treatment intent as a stratification factor, participants were randomly allocated 1:1 to nonintervention or intervention groups, in which pharmacists delivered only standard medication counseling as in usual practice or additionally provided structured information regarding CGP, respectively. The primary endpoint was the proportion of patients demonstrating reduced Decisional Conflict Scale (DCS) scores before and after pharmacist interviews, 2–6 weeks follow-up interval. Secondary endpoint analysis explored factors associated with patient decision making. Data from 180 patients were included in the final analysis. The proportion of patients with decreased DCS scores did not differ significantly between the intervention and non-intervention groups (49.4% vs. 41.9%; *p* = 0.31). However, while decisional conflict regarding treatment selection increased in both groups, the amplitude of this increase was significantly lower in the pharmacist intervention group, with mean DCS scores rising by only 1.98 ± 16.5 compared to 3.61 ± 17.3 in the non-intervention group (*p* = 0.0261). In conclusion, although early pharmacist-led information provision did not change the overall proportion of improved patients, it successfully limited the rise of decisional conflict amplitude. These findings demonstrate that pharmacists can play an active, valuable role in supporting patients for cancer genome medicine-related patient decisions.

## 1 Introduction

Cancer is a leading cause of mortality worldwide, accounting for ∼10 million deaths as of 2022.^1^ In Japan, approximately 380,000 individuals died from cancer in 2023,^2^ primarily due to lung, colorectal, and pancreatic cancers, with disease progression from distant metastases representing the major cause of mortality.^3^ Notably, hereditary cancer predisposition syndromes contribute to an estimated 5%–10% of all cancer cases.^4^

The principal cancer treatments include surgery and radiation therapy as local modalities, along with drug therapy as a systemic approach. Depending on disease stage, these treatments may be administered alone or in combination through multidisciplinary care. However, for advanced recurrence or widespread disease, drug therapy represents the primary treatment option. Because drug therapy rarely cures metastatic, recurrent, or advanced solid tumors, improving therapeutic outcomes remains a central challenge in oncology. One strategy to address this challenge is comprehensive genome profiling (CGP).

In November 2017, CGP was introduced in the United States to comprehensively characterize tumor mutation profiles in patients with advanced recurrent solid tumors.^5^ The American Society of Health-System Pharmacists has published a statement outlining the pharmacist's role in clinical pharmacogenomics,^6^ recommending drug selection based on pharmacogenomic testing results. The statement emphasizes the importance of information provision and patient empowerment to support testing and result interpretation. Although the role of pharmacists in precision oncology within multidisciplinary teams has been described,^7^ patient-facing involvement for individuals considering CGP is limited. While it has been reported that improved health literacy reduces decision-making conflict,^8^ previous studies have not tested pharmacist-led education in genomic medicine decision making. Therefore, examining the role of pharmacists in providing information is a novel approach.

In Japan, CGP became insurance-covered in June 2019, and by 2025, cancer genome medicine was at 282 institutions designated by the Ministry of Health, Labour, and Welfare.^9^ Institutions without designation cannot provide cancer genomic medicine, limiting nationwide dissemination and awareness. Because CGP is intended for patients who have completed standard treatment or have rare cancers, many patients hold high expectations of therapeutic benefit.^10^ However, actionable genetic alterations are not always identified, and even when candidate drugs are found, they may not be approved or available in Japan. Currently, only an estimated 10%–15% of patients undergoing CGP ultimately receive treatment based on the results.^11^

Information regarding test precautions is available through patient-oriented websites and other sources; however, it remains unclear whether patients access or correctly understand this information. A 2023 Cabinet Office public opinion survey on cancer control reported that most respondents (56.2%) identified physicians and nurses at hospitals or clinics as primary information sources following a cancer diagnosis, followed by family, friends, and acquaintances (36.7%) and then the Internet (26.2%).^12^ These findings suggest that patients largely depend on healthcare professionals involved in their care for reliable information.

Japanese pharmacists frequently interact directly with patients with cancer.^13^ Pharmacists provide chemotherapy explanations for both inpatients and outpatients and engage with patients in community pharmacies. Therefore, they may serve as accessible resources when patients seek information about CGP. Drawing on the Ottawa Decision Support Framework,^14^ we hypothesized that providing accurate CGP information before testing decisions may improve patient health literacy and reduce decisional conflict about future genomic testing, about current treatment, or about overall treatment trajectory. Accordingly, we examined whether pharmacist-led CGP information provided earlier than the completion of standard treatment influences patient decision making.

Decisional conflict is commonly assessed using the decisional conflict scale (DCS) developed by O'Connor et al.15., 16., 17., 18., 19., 20., 21., 22., 23., 24., 25., 26., 27., 28., 29. The DCS includes 16 items scored on a 0–100 scale, with lower scores indicating less decisional conflict. CGP testing is characterized by uncertainty regarding results, potential impact on families,^30^ and a low probability of leading to treatment.^11^ For these reasons, we considered that DCS is suitable for CGP decisions. In the present study, DCS scores were recorded before and after pharmacists provided information to quantitatively evaluate changes in patient perspectives. Secondary analyses were conducted to identify factors associated with changes in decisional attitudes.

## 2 Methods

### 2.1 Study design

This is an open-label, randomized, controlled study enrolled patients who received chemotherapy at Juntendo University Hospital, Tokyo, Japan, between March 2022 and July 2023.

### 2.2 Eligibility criteria

Eligibility criteria included age ≥ 20 years, receipt of standard insurance-covered care, and potential eligibility for CGP after completion or anticipated completion of standard treatment. Potential eligibility for CGP was determined by the treating physician, based on the patient's overall clinical condition, including performance status, organ function, and the likelihood of being able to receive genome-matched therapy upon result disclosure. Patients who had difficulty making independent decisions owing to their psychiatric symptoms or cognitive impairment were excluded by a clinical examination.

### 2.3 Sample size

Sample size was calculated for the primary endpoint, defined as the between-group difference in the proportion of patients showing a decrease in DCS scores before and after the pharmacist interview. Because no formal pilot data were available, we assumed a small effect size of 0.2 as a conservative design value. This assumption was informed by prior DCS literature and the DCS user manual, which indicates that clinically relevant intervention effects are often in the small-to-moderate range, with sample size planning commonly based on effect sizes around 0.30–0.40 and some between-intervention differences reported around 0.2–0.3. Given that our intervention was a brief pharmacist-led informational support program rather than a more intensive decision aid, we considered a small effect size to be a reasonable and conservative assumption. On this basis, 88 patients per group were estimated to provide 80% power with a two-sided significance level of 0.05.^31^

### 2.4 Randomization

Patients were randomly assigned 1:1 to the intervention or nonintervention group, stratified by treatment objective (preoperative/postoperative vs. advanced recurrence). The allocation sequence was computer-generated, and staff responsible for randomization obtained group allocation codes from the Medical Technology Innovation Center Clinical Research and Trial Center, Juntendo University. Block randomization was not used. Pharmacists were not blinded and could know treatment allocation before completion of patient enrollment. Therefore, allocation concealment was not implemented. Owing to the nature of the intervention, blinding of pharmacists and participants after assignment was also not feasible.

### 2.5 Interventions

In the nonintervention group, pharmacists provided medication explanations consistent with usual care in the outpatient chemotherapy unit. In contrast, patients in the intervention group additionally received general information on cancer genome medicine using a patient handbook [32, Supplemental material 1] as an anticipatory education, including details on eligibility and available facilities. The intervention group was also guided to the Center for Cancer Genomics and Advanced Therapeutics website designed for patients.

Five pharmacists, with a mean of 14.8 ± 5.8 years of professional experience, participated in providing cancer genome medicine information. All pharmacists were certified in cancer chemotherapy and had received training in cancer genome medicine. The duration of each pharmacist interview was recorded.

### 2.6 Data collection

Patient demographic data collected included age, gender, cancer type, family history, disease classification, treatment line, history of nonsurgical treatments [chemotherapy, radiofrequency ablation (RFA), transcatheter arterial chemoembolization (TACE), and microwave ablation (MWA)], marital status, presence of children, employment status, highest education level, income, and sources of medical information. Questionnaires were administered 2–6 weeks before and after the pharmacist interview to assess DCS scores, which were calculated according to the user manual. The 2–6-week interval was selected because many outpatients receive chemotherapy every 2–3 weeks.

After the pharmacist interview, patients were asked whether they wished to undergo CGP and which information sources informed their decision. Patients were also asked how much they would be willing to pay for the provision of cancer genome medicine information.

### 2.7 Study outcomes

The primary endpoint was proportion of patients with a decrease in DCS scores. As a secondary endpoint, relationships between patient factors influencing decision making and DCS scores were analyzed.

### 2.8 Statistical analysis

The proportion of patients with decreased DCS scores before and after pharmacist interviews was calculated. A test of proportions was used to compare changes between the intervention and nonintervention groups. Mean changes in DCS scores before and after the pharmacist interview were also calculated and compared between groups using an unpaired *t*-test. These were carried out as a predefined analysis. As an exploratory analysis, a one-way analysis of covariance (ANCOVA) was conducted to evaluate the effectiveness of the intervention program (intervention vs. nonintervention group) on post DCS scores while controlling for baseline scores as a covariate. Prior to the analysis, the assumptions of normality, homogeneity of variances, and homogeneity of regression slopes were verified.

Baseline DCS scores were divided into two groups at the median to examine trends in patient background characteristics. In the intervention group, patients were further divided into those with decreased versus increased DCS scores, and background trends were similarly evaluated. Age differences between groups were analyzed using a *t*-test. Associations between patient factors influencing decision making and DCS scores were analyzed using the chi-square test or Fisher's exact test. Secondary and subgroup analyses were exploratory in nature and were conducted to identify potential patterns associated with patient decision making. No formal adjustment for multiple testing was applied; therefore, results from these analyses should be interpreted as hypothesis-generating. Statistical analyses were performed using Statistical Analysis Software (SAS) (v9.4; SAS Institute Inc., Cary, North Carolina) and R (version 2026.01.1 + 403; R Foundation for Statistical Computing, Vienna, Austria).

### 2.9 Registration

This study was conducted in accordance with the ethical guidelines for life science and medical research involving human subjects and the Declaration of Helsinki. Written informed consent was obtained from all participants. The study was approved by Research Ethics Committee of the Faculty of Medicine, Juntendo University (E21–0016), and registered in Japan Registry of Clinical Trials (jRCT1030210390).

## 3 Results

**Fig. 1:**
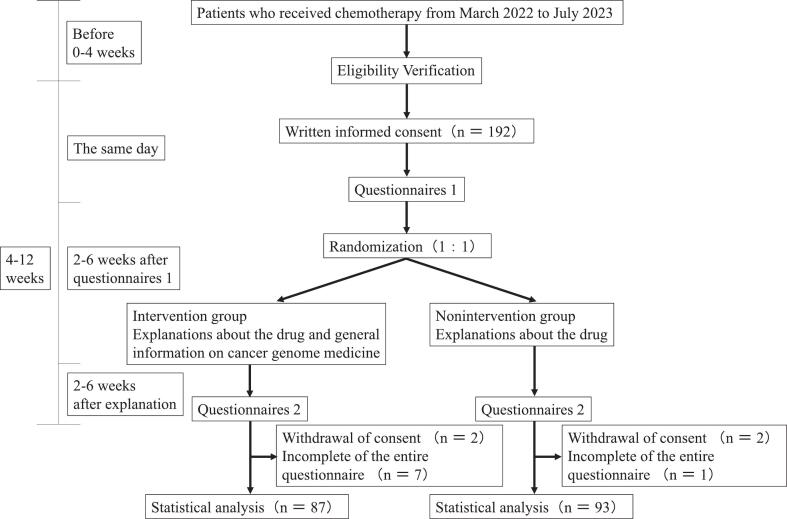
Outline of study

Written consent was obtained from 192 patients who received chemotherapy at Juntendo University Hospital during March 2022–July 2023. Of these, four patients withdrew consent and eight were unable to complete the full questionnaire. Thus, responses from 180 patients were included in the final analysis (Fig. 1). There were not treatment changes or clinical milestones during the follow-up.

Patient characteristics were evaluated to determine whether background factors that could influence genome-related treatment decision making differed between groups (Table 1). No significant differences in baseline characteristics were observed between the intervention and nonintervention groups. Patient sources of medical information were also examined. The most frequently reported source was the Internet, followed by friends and acquaintances (Table 1).

**Table 1:** Patient background of all patients.

		Intervention		Non-intervention		*p* value
		Mean	(SD)	Mean	(SD)	
Age		56.8	(10.1)	57.1	(10.9)	0.840
		n	(%)	n	(%)	
Gender		87	(100.0)	93	(100.0)	0.536⁎
	Males	28	(32.2)	26	(28.0)	
	Females	59	(67.8)	67	(72.0)	
Type of cancer						ー
	Gastric cancer	6	(6.9)	4	(4.3)	
	Esophageal cancer	1	(1.1)	3	(3.2)	
	Colorectal cancer	8	(9.2)	5	(5.4)	
	Lung cancer	10	(11.5)	11	(11.8)	
	Breast cancer	30	(34.5)	35	(37.6)	
	Liver cancer	2	(2.3)	7	(7.5)	
	Biliary cancer	1	(1.1)	1	(1.1)	
	Pancreatic cancer	9	(10.3)	10	(10.8)	
	Endometrial cancer	8	(9.2)	9	(9.7)	
	Cervical cancer	2	(2.3)	2	(2.2)	
	Ovarian cancer	10	(11.5)	6	(6.5)	
Stage						0.543⁎
	1	17	(19.5)	15	(16.1)	
	2	19	(21.8)	25	(26.9)	
	3	27	(31.0)	22	(23.7)	
	4	24	(27.6)	31	(33.3)	
Purpose of treatment						0.809⁎
	preoperative	12	(13.8)	14	(15.1)	
	postoperative	34	(39.1)	32	(34.4)	
	advanced recurrence	41	(47.1)	47	(50.5)	
Lines of chemotherapy						0.498†
	1st	54	(62.1)	62	(66.7)	
	2nd	26	(29.9)	28	(30.1)	
	3rd	3	(3.4)	2	(2.2)	
	4th	1	(1.1)	1	(1.1)	
	others	3	(3.4)	0	(0.0)	
History of non-surgical treatment						0.458⁎
	Yes	38	(43.7)	36	(38.7)	
	No	49	(56.3)	57	(61.3)	
Family history						0.908⁎
	Yes	25	(28.7)	26	(28.0)	
	No	62	(71.3)	67	(72.0)	
Marital status						0.550⁎
	Married	21	(24.1)	19	(20.4)	
	Others	66	(75.9)	74	(79.6)	
Child(ren)						0.059⁎
	Yes	45	(51.7)	61	(65.6)	
	No	42	(48.3)	32	(34.4)	
Occupation						0.070†
	Full-time	55	(63.2)	45	(48.4)	
	Part-time	8	(9.2)	18	(19.4)	
	Student	0	(0.0)	0	(0.0)	
	Unemployed	24	(27.6)	30	(32.3)	
Education						0.607†
	Junior high school	3	(3.4)	4	(4.3)	
	High school	19	(21.8)	20	(21.5)	
	Occupational school	20	(23.0)	13	(14.0)	
	Junior college	10	(11.5)	13	(14.0)	
	University or graduate school	35	(40.2)	43	(46.2)	
Annual household income (×10^6^ yen)						ー
	0	4	(4.6)	4	(4.3)	
	−0.99	2	(2.3)	0	(0.0)	
	1.00–1.99	6	(6.9)	8	(8.6)	
	2.00–2.99	9	(10.3)	6	(6.5)	
	3.00–3.99	11	(12.6)	12	(12.9)	
	4.00–4.99	9	(10.3)	14	(15.1)	
	5.00–9.99	29	(33.3)	32	(34.4)	
	10.00–14.99	13	(14.9)	10	(10.8)	
	15.00-	4	(4.6)	7	(7.5)	
Sources of medical information (multiple choice)						ー
	Families and friends	45	(51.7)	56	(60.2)	
	Newspaper	22	(25.3)	24	(25.8)	
	Magazines	12	(13.8)	12	(12.9)	
	Books	12	(13.8)	13	(14.0)	
	TV/Radio programs	38	(43.7)	45	(48.4)	
	Web	61	(70.1)	62	(66.7)	
	Public agencies	22	(25.3)	21	(22.6)	
	Medical institution	44	(50.6)	47	(50.5)	
	Pharmaceutical company	14	(16.1)	25	(26.9)	
	Individuals	26	(29.9)	21	(22.6)	
	Social network service	19	(21.8)	10	(10.8)	
	Lectures	1	(1.1)	2	(2.2)	
	Others	5	(5.7)	6	(6.5)	

SD; standard deviation, n; Number of patients.

Percentages are rounded to one decimal place; therefore, totals may not sum to exactly 100%.

⁎ Chi-square test.

† Fisher's exact test.

The mean durations of pharmacist interviews were 19.9 ± 6.9 and 10.4 ± 4.3 min in the intervention and nonintervention groups, respectively. Following the pharmacist interview, DCS scores decreased in 49.4% and 41.9% of patients in the intervention and nonintervention groups, respectively; this difference was not statistically significant (*p* = 0.31, 95% CI: −7.0 to 22.0 percentage points, test of proportions; Table 2). In contrast, mean DCS scores increased by 1.98 ± 16.5 and 3.61 ± 17.3 in the intervention and nonintervention groups, respectively (95% CI: −6.60 to 3.34 points, Table 2).

**Table 2:** Difference of Decisional Conflict Scale (DCS) score between before and after the pharmacist interview.

	Intervention		Non-intervention		*p* value
	n	(%)	n	(%)	
Difference of DCS score < 0	43	(49.4)	39	(41.9)	0.3133
Difference of DCS score ≧0	44	(50.6)	54	(58.1)	
	Mean	(SD)	Mean	(SD)	
Difference of DCS score	1.98	(16.5)	3.61	(17.3)	0.0261

n; Number of patients, SD; standard deviation.

The mean baseline DCS score was 39.3 and 38.4 in the intervention and nonintervention groups, respectively. The mean post DCS score was 41.3 and 42.0 in the intervention and nonintervention groups, respectively. In an ANCOVA adjusted for baseline DCS scores and stratification factors, the adjusted mean post DCS score was 41.1 and 42.2 in the intervention and nonintervention groups, respectively. The difference in adjusted means between the intervention and control groups was 1.10 points, with a 95% confidence interval of −3.16 to 5.36 and a *p*-value of 0.612. Therefore, no clear evidence was found to indicate that the intervention reduced decision-making conflict.

Baseline DCS scores were strongly associated with post DCS scores (regression coefficient 0.49, 95% confidence interval 0.37 to 0.61, *p* < 0.001). In contrast, no clear association was observed between stratification factors and the post DCS scores. In a sensitivity analysis that further adjusted for age and gender, the estimated intervention effect remained similar and was not statistically significant (adjusted mean difference 1.20 points, 95% confidence interval − 3.04 to 5.43, *p* = 0.579). Furthermore, in a sensitivity analysis using the change in DCS scores as the outcome, the difference between groups was not clear (mean difference 1.60 points, 95% confidence interval − 3.40 to 6.59, *p* = 0.529). Moreover, even when using the heteroskedasticity-consistent 3 robust standard error, there was no substantial change in the conclusions.

Background factors influencing treatment decisions, including CGP, were further analyzed. Patients were divided into two groups according to the median DCS score before the pharmacist interview, and background characteristics were compared between groups (Supplemental Data Table 1). No variables showed significant difference between the groups.

Factors associated with decision making after the pharmacist intervention were subsequently assessed. The intervention group was subdivided into patients with decreased versus increased DCS scores following the interview, and background characteristics were compared (Table 3). Patients with decreased DCS scores tended to be younger than those with increased scores (*p* = 0.024). In the decreased-score group, 21 and 18 patients were receiving primary and secondary treatment, respectively. In comparison, the increased-score group included 33 patients undergoing primary treatment and a higher number of patients without a history of nonsurgical treatments, e.g., chemotherapy, RFA, TACE, and MWA (31 patients).

**Table 3:** Patient background of intervention group when they were divided into two groups (those whose DCS scores decreased and increased after the interview).

		Difference of DCS score < 0		Difference of DCS score ≧0		*p* value
		Mean	(SD)	Mean	(SD)	
Age		54.37	(10.3)	59.25	(9.5)	0.024
		n	(%)	n	(%)	
Gender		43	(100.0)	44	(100.0)	0.594⁎
	Males	15	(34.9)	13	(29.5)	
	Females	28	(65.1)	31	(70.5)	
Type of cancer						ー
	gastric cancer	5	(11.6)	1	(2.3)	
	esophageal cancer	1	(2.3)	0	(0.0)	
	colorectal cancer	5	(11.6)	3	(6.8)	
	lung cancer	5	(11.6)	5	(11.4)	
	breast cancer	16	(37.2)	14	(31.8)	
	liver cancer	1	(2.3)	1	(2.3)	
	biliary cancer	1	(2.3)	0	(0.0)	
	pancreatic cancer	3	(7.0)	6	(13.6)	
	endometrial cancer	2	(4.7)	6	(13.6)	
	cervical cancer	2	(4.7)	0	(0.0)	
	ovarian cancer	2	(4.7)	8	(18.2)	
Stage						0.755⁎
	1	9	(20.9)	8	(18.2)	
	2	10	(23.3)	9	(20.5)	
	3	11	(25.6)	16	(36.4)	
	4	13	(30.2)	11	(25.0)	
Purpose of treatment						0.665
	preoperative	7	(16.3)	5	(11.4)	
	postoperative	15	(34.9)	19	(43.2)	
	advanced recurrence	21	(48.8)	20	(45.5)	
Lines of chemotherapy						0.035†
	1st	21	(48.8)	33	(75.0)	
	2nd	18	(41.9)	8	(18.2)	
	3rd	2	(4.7)	1	(2.3)	
	4th	0	(0.0)	1	(2.3)	
	others	2	(4.7)	1	(2.3)	
History of non-surgical treatment						0.007⁎
	Yes	25	(58.1)	13	(29.5)	
	No	18	(41.9)	31	(70.5)	
Family history						0.533⁎
	Yes	31	(72.1)	29	(65.9)	
	No	12	(27.9)	15	(34.1)	
Marital status						0.235⁎
	Married	24	(55.8)	30	(68.2)	
	Others	19	(44.2)	14	(31.8)	
Child(ren)						0.745⁎
	Yes	23	(53.5)	22	(50.0)	
	No	20	(46.5)	22	(50.0)	
Occupation						0.330†
	Full-time	25	(58.1)	30	(68.2)	
	Part-time	6	(14.0)	2	(4.5)	
	Student	0	(0.0)	0	(0.0)	
	Unemployed	12	(27.9)	12	(27.3)	
Education						0.737†
	Junior high school	2	(4.7)	1	(2.3)	
	High school	8	(18.6)	11	(25.0)	
	Occupational school	9	(20.9)	11	(25.0)	
	Junior college	4	(9.3)	6	(13.6)	
	University or graduate school	20	(46.5)	15	(34.1)	
Annual household income (×10^6^ yen)						0.890†
	0	3	(7.0)	1	(2.3)	
	−0.99	0	(0.0)	2	(4.5)	
	1.00–1.99	3	(7.0)	3	(6.8)	
	2.00–2.99	4	(9.3)	5	(11.4)	
	3.00–3.99	5	(11.6)	6	(13.6)	
	4.00–4.99	5	(11.6)	4	(9.1)	
	5.00–9.99	15	(34.9)	14	(31.8)	
	10.00–14.99	7	(16.3)	6	(13.6)	
	15.00-	1	(2.3)	3	(6.8)	
Sources of medical information (multiple choice)						ー
	Families and friends	22	(51.2)	23	(52.3)	
	Newspaper	9	(20.9)	13	(29.5)	
	Magazines	8	(18.6)	4	(9.1)	
	Books	6	(14.0)	6	(13.6)	
	TV/Radio programs	19	(44.2)	19	(43.2)	
	Web	30	(69.8)	31	(70.5)	
	Public agencies	10	(23.3)	12	(27.3)	
	Medical institution	21	(48.8)	23	(52.3)	
	Pharmaceutical company	11	(25.6)	3	(6.8)	
	Individuals	13	(30.2)	13	(29.5)	
	Social network service	10	(23.3)	9	(20.5)	
	Lectures	0	(0.0)	1	(2.3)	
	Others	3	(7.0)	2	(4.5)	

SD; standard deviation, n; Number of patients.

Percentages are rounded to one decimal place; therefore, totals may not sum to exactly 100%.

⁎ Chi-square test.

† Fisher's exact test.

Patients were also asked whether they wished to undergo CGP after the pharmacist interview. No significant differences were observed between the two groups (Supplemental Data Table 2). Table 4 shows the information sources referenced when patients expressed their preferences. In the intervention group, most respondents (61) cited explanations provided by pharmacists, followed by information obtained from the website (26 respondents). In the nonintervention group, the website was the most frequently cited source of information (34 respondents).

**Table 4:** Sources of information used as a reference when responding to whether or not a desire to take a CGP after the pharmacist interview (multiple choice).

	Intervention		Non-intervention	
	n	(%)	n	(%)
	87		93	
Families and friends	7	(8.0)	13	(14.0)
Newspaper	5	(5.7)	8	(8.6)
Magazines	5	(5.7)	2	(2.2)
Books	1	(1.1)	2	(2.2)
TV/Radio programs	8	(9.2)	8	(8.6)
Web	26	(29.9)	34	(36.6)
Public agencies	9	(10.3)	8	(8.6)
Medical institution	13	(14.9)	20	(21.5)
Pharmaceutical company	9	(10.3)	12	(12.9)
Individuals	2	(2.3)	2	(2.2)
Social network service	3	(3.4)	1	(1.1)
Lectures	2	(2.3)	0	(0.0)
Pharmacist	61	(70.1)	24	(25.8)
Others	10	(11.5)	21	(22.6)

n; Number of patients.

Willingness to pay for information provision was additionally assessed. In both groups, most respondents reported a willingness to pay 600–1000 yen (∼4–6 USD), followed by those willing to pay ≥1000 yen (≥6 USD). No significant differences were identified between the intervention and nonintervention groups (Supplemental Data Table 3). As a reference, a total score and sub scores of DCS are shown (Supplemental Data Table 4).

## 4 Discussion

DCS scores increased in both groups. Although the increase appeared smaller in the intervention group, this difference was not statistically significant after adjustment for baseline values. Increased DCS scores reflect heightened decisional conflict regarding treatment selection, including CGP, regardless of whether patients ultimately wished to receive treatment. Although only the intervention group received detailed information on CGP, the nonintervention group was exposed to the term “CGP” during consent procedures. Exposure to unfamiliar terminology may have caused hesitation by introducing new potential treatment concepts. In this study, we did not examine what actions were taken after the pharmacist interview, also we did not restrict patients from collecting information on their own. This survey revealed that a high percentage of respondents relied on the Internet and media, along with family and friends, for medical information. If patients obtained some information from the Internet and media, it may contribute to uncertainty. With the expansion of online resources, including social media, patients can easily access diverse information; however, such information is often more ambiguous than that obtained from traditional media (e.g., TV and newspapers), underscoring the importance patient information literacy.

On the other hand, in the intervention group, the pharmacist interview might have led participants to learn about CGP as a new treatment option, potentially causing them to question whether their current treatment was optimal. Furthermore, recognizing the uncertainty that the results of CGP might not necessarily lead to treatment could have increased their internal conflict.

We compared the proportion of patients with decreased versus non-decreased DCS scores rather than the magnitude of score change. Because changes in score magnitude alone do not capture the direction of change. The proportion of patients with decreased DCS scores after the pharmacist interview was higher in the intervention group than in the nonintervention group, although the difference was nonsignificant. DCS scores were measured 2–6 weeks before and after the pharmacist interview to support reliable questionnaire responses. Nevertheless, DCS scores are highly time-dependent, and the limited period available for decision making may have reduced the ability to detect significant differences.

In the intervention group, more patients cited the pharmacist's explanation when deciding whether to undergo CGP. These patients received information on eligibility, potential outcomes, and broader perspectives related to CGP. This finding highlights the value of combining information from media and online sources with bidirectional communication between patients and pharmacists, who represent highly reliable information providers. The pharmacist interview required ∼10 additional minutes in the intervention group, suggesting that CGP-related information can be delivered within a feasible timeframe. It is a new and valuable reveal that this duration appears reasonable and practical for implementation in medical practice. The intervention group had a longer pharmacist interview time than the nonintervention group. Although this is not a confounder in the strict sense because allocation was randomized, the additional contact time itself may have influenced patient-reported decisional conflict. Therefore, part of the observed effect may reflect greater attention or supportive communication rather than the informational content alone.

To identify patients experiencing decisional conflict regarding treatment, including CGP consultation, demographic characteristics were analyzed after dividing patients according to the median baseline DCS score. No differences were observed between groups, indicating no clear characteristics associated with greater or lesser decisional conflict. Although health care providers often consider factors such hereditary cancer or family history when anticipating decisional difficulty, these findings suggest that CGP information provision should not be limited to specific patient subgroups. In the present study, the median was used to assess patient tendencies; however, identifying patients who require targeted intervention may require alternative cutoffs, such as high (e.g., 80) or low (e.g., 20) DCS scores.

To further explore factors associated with changes in decisional conflict, the intervention group was divided into patients with decreased versus increased DCS scores after the pharmacist interview. Younger age was more common among patients with decreased DCS scores, suggesting that pharmacist-provided information may be particularly effective in younger patients. Patients receiving first-line chemotherapy tended to show increased DCS scores, possibly because they are generally ineligible for CGP in Japan. Information regarding CGP may have created the impression of additional treatment options, thereby causing confusion. Conversely, some patients receiving first- or second-line chemotherapy showed decreased DCS scores, suggesting that pharmacist explanations may have reassured them that their current treatment was appropriate for their disease stage. An increase in DCS scores was also observed among patients without a history of nonsurgical treatment. Collectively, these findings indicate that although early, future-oriented information may benefit some patients, uniform information provision may increase confusion in others and should be applied cautiously. Several subgroup and secondary analyses were performed in this study, and no formal adjustment for multiple testing was applied. Accordingly, these findings should be interpreted as exploratory and hypothesis-generating rather than confirmatory. In particular, nominally significant associations observed in subgroup analyses may represent chance findings and should be validated in future studies.

No significant differences were observed between groups in the proportion of patients wishing to undergo CGP. Approximately 58.6% and 54.8% of patients in the intervention and nonintervention groups, respectively, answered no, whereas 26.4% and 30.1% of patients in these respective groups did not respond. These nonresponses likely included patients who were indecisive or who concluded that CGP was not appropriate at the time.

Regarding willingness to pay for information, most respondents in both groups selected 600–1000 yen (∼4–6 USD), followed by ≥1000 yen (≥6 USD). Under Japan's universal health insurance system, pharmacists currently charge 600–1000 yen for chemotherapy-related counseling. The finding that many patients were willing to pay ≥1000 yen implies that they value high-quality information provision.

The present study was powered for the primary endpoint only and was not specifically designed to detect modest but clinically meaningful differences in secondary outcomes. Accordingly, the secondary analyses should be regarded as exploratory and hypothesis-generating. In particular, the absence of statistically significant differences in secondary endpoints should not be interpreted as firm evidence of no effect, because limited sample size may have reduced the ability to detect smaller but potentially important differences. Future studies with larger sample sizes and prespecified secondary endpoint hypotheses are needed to confirm these findings.

Another statistical limitation is that continuous DCS scores change was compared using a simple *t*-test rather than a model adjusting for baseline DCS score. An ANCOVA approach may have provided a more efficient estimate of the between-group difference by accounting for baseline variation. Because this approach was not prespecified in the original analysis plan, we retained the original analysis for the present report. Future studies should consider prespecifying baseline-adjusted models for continuous DCS scores outcomes. A further methodological consideration is that the primary endpoint dichotomized DCS scores change into decreased (<0) versus non-decreased (≥0). Although this approach captures the direction of change, dichotomization may reduce statistical power and may obscure potentially meaningful differences in the magnitude of change. In the present study, this endpoint was prespecified and used for sample size determination; therefore, it was retained as the primary analysis. However, the continuous change in DCS score was also analyzed as a complementary outcome, and future studies may benefit from prespecifying continuous DCS scores change as the primary endpoint to improve statistical efficiency and interpretability.

The primary role of pharmacists in cancer care is to support drug therapy and manage adverse effects.^29^ In cancer genome medicine, CGP results are used to identify potential therapies and clinical trial options as well as support drug administration.5., 6. However, because many CGP-based treatments in Japan are conducted within clinical trials, pharmacist involvement is often limited to specific medical institutions.33., 34. In this study, we explored the role of pharmacists from a completely new perspective, different from conventional approaches. Providing information about CGP testing by pharmacists did not significantly reduce DCS scores. However, the fact that DCS scores increased in both groups suggests that explanations regarding CGP testing may have caused patients to recognize new uncertainties and brought decision-making conflict to the surface. We consider that this uncertainty includes both the possibility that CGP may offer a new treatment option and the possibility that no treatment based on the results of CGP may exist. This finding may reflect a transition from uninformed certainty to informed uncertainty. Therefore, when explaining CGP testing, it is important not only to provide information but also to consider the timing of the explanation, allow patients time to reflect, and offer ongoing decision-making support. Pharmacists possess unique competencies in translating intricate scientific data such as pharmacogenetic profiles into actionable, patient-centered language. By effectively explaining the clinical relevance of genetic information (e.g., how specific gene variants impact their individual drug efficacy or side-effect risks), pharmacists may support informed decision making despite persistent uncertainty and enhance their “informed choice.” We consider that this specific translational communication skill may become a key factor in reducing conflict. By developing a solid understanding of CGP and its therapeutic implications, pharmacists can provide timely, patient-centered information and contribute to future cancer care. Although we consider that pharmacists may help reduce confusion from complex tests, it could not be clearly stated that pharmacists are helpful because conflicts increased in this study. Similarly, the 49.4% improvement may reflect a positive aspect of the usefulness of pharmacists, but it is possible that the study was too small to detect it. By reviewing the research design, it may be possible to demonstrate the usefulness of pharmacists, which remains a challenge for the future.

This study was conducted at a single center in Japan, which may limit generalizability. Differences in access to genomic medicine facilities, such as between urban and rural regions, may also influence patient characteristics and outcomes. Furthermore, in countries where most citizens are covered by health insurance systems and have access to genetic panel testing under public coverage, such as Germany and South Korea,35., 36. the timing of CGP testing is not restricted to after the completion of standard treatment.^37^ These differences suggest that international comparisons may help inform the optimal timing of information provision in future research.

This study has several limitations. The small sample size and short interval length may have reduced statistical power and limited the detection of significant differences. As another possibility, this study was designed to evaluate whether pharmacists providing CGP information earlier than the completion of standard therapy affects patient decision making, which was our primary objective. The results this study may indicate not that the effect of the pharmacist was limited, but rather that the primary endpoints may not have had sufficient power to verify our objectives. In addition, as an open-label study and relies on self-reported outcomes, the potential influence of the Hawthorne effect cannot be ruled out. Without fidelity assessment to the intervention, variability among pharmacists might confound results. This study includes no assessment of actual CGP uptake or clinical outcomes. Moreover, there is a possibility of contamination because the patients were not restricted from accessing online resources. Although randomization was stratified by treatment objective to account for differences between perioperative and advanced/recurrent disease settings, decisional conflict may also be influenced by disease severity, symptom burden, social support, health literacy, and health system-related factors. These factors were not comprehensively measured or adjusted for; therefore, residual confounding cannot be excluded. Furthermore, this study has another limitation that need to be addressed in future research. Specifically, the potential for bias from unmeasured or unknown confounders—such as simultaneous support from other health professionals like nurses or genetic counsellors who could produce similar outcomes—needs further study.

## 5 Conclusions

Pharmacist-provided information did not alter short-term patient decision making. While CGP testing brought awareness of uncertainty for everyone, the pharmacist intervention was associated with a significantly smaller increase in conflict. Therefore, pharmacists can demonstrate their value by using clear communication to tailor support to the optimal timing of the intervention, the patient's baseline understanding, and their conflict. In the next step, we will examine strategies for tailoring pharmacist-led information provision by appropriately narrowing the target patient subgroups and timing, thereby minimizing the potential for patient confusion.

### 5.1 Practice implications

Although patients have access to extensive medical information, insufficient support may increase decisional conflict. Pharmacists frequently interact with patients with cancer and possess expertise in drug therapy. From the perspective of post-CGP treatment, pharmacists can offer guidance at different times and from perspectives distinct from doctors. Such support may help patients make more informed decisions with greater understanding.

## Author contributions: CRediT

Orie Saigo: Writing – original draft, conceptualization, methodology, software, formal analysis, resources, data Curation, visualization, supervision, project administration. Shuko Nojiri: Methodology, formal analysis, validation, writing – review & editing. Kota Asakura: Investigation, resources. Shoji Koshiba: Investigation, resources. Uki Saito: Investigation, resources. Satoshi Iwakawa: Investigation, resources. Toshimi Kimura: Writing – review & editing. Shunsuke Kato: Writing – review & editing, methodology, project administration, funding acquisition.

## CRediT authorship contribution statement

**Orie Saigo:** Writing – original draft, Visualization, Supervision, Software, Resources, Project administration, Methodology, Investigation, Formal analysis, Data curation, Conceptualization. **Shuko Nojiri:** Validation, Methodology, Formal analysis. **Kota Asakura:** Resources, Investigation. **Shoji Koshiba:** Resources, Investigation. **Uki Saito:** Resources, Investigation. **Satoshi Iwakawa:** Resources, Investigation. **Toshimi Kimura:** Writing – review & editing. **Shunsuke Kato:** Writing – review & editing, Project administration, Methodology, Funding acquisition.

## Declaration of generative AI and AI-assisted technologies in the writing process

During the preparation of this work, the author used generative AI tools to assist with language refinement. The author takes full responsibility for the content of this work and has carefully reviewed and edited all outputs generated by the AI.

## Funding

This study was supported, in part, by Daiichi Sankyo Scholarship Donation Program. The funder had no role in design, analysis, interpretation.

## Declaration of competing interest

The authors declare no conflicts of interest associated with this manuscript.

## Data Availability

The deidentified data that support the findings of this study are available by request from the corresponding author.

## Appendix A Supplementary data

Supplementary material: Supplemental data Table 1. Patient background of all patients when they were divided into two groups based on the median DCS score before the pharmacist interview Supplemental data Table 2. Patients were asked about their desire to take a CGP after the pharmacist interview Supplemental data Table 3. How much patients would be willing to pay for information Supplemental data Table 4. Total score and sub scores of DCS
